# Study on the energy evolution mechanism of coal and rock with impact tendency under different strain rates

**DOI:** 10.1038/s41598-023-41094-5

**Published:** 2023-08-23

**Authors:** Kun Zhang, Yichen Zhang, Sen Zhang, Jianxi Ren, Liang Zhang, Renjie Zhang, Yuanquan Cui

**Affiliations:** School of Architecture and Civil Engineering, Xi’an University of Science and Technology, Xi’an, 710054 China

**Keywords:** Civil engineering, Materials science

## Abstract

To explore the strain rate effect of deformation and failure of impact prone coal rock, uniaxial compression tests and triaxial compression tests with different strain rates were carried out. The mechanical properties and impact tendency of impact-prone coal rock were studied, and the energy evolution law and pre-peak energy self-promotion-inhibition mechanism of impact-prone coal rock were obtained. The results show that with the increase of strain rate, the peak strength of coal rock under uniaxial compression decreases gradually, and the peak strength of coal rock under triaxial compression increases first and then decreases, and the impact tendency of coal rock increases first and then decreases. The energy evolution of coal rock under uniaxial compression is mainly divided into four stages: initial energy damage, energy hardening, energy softening and failure. With the increase of strain rate, the total energy and elasticity at the peak point of coal rock under uniaxial compression decrease gradually, and the total energy, elastic energy and dissipation energy at the peak point under triaxial compression increase first and then decrease. The elastic energy promotion coefficient of impact-prone coal rock is much larger than the inhibition coefficient, and the increase of strain rate will promote the generation of elastic energy inside coal rock. The research results can provide reference for the prevention and early warning of dynamic disasters of coal and rock mass with impact tendency.

## Introduction

In recent years, with the further development of deep coal mining, the frequency and scale of rock burst disasters in coal mines have increased significantly. The internal structure of coal rock is highly complex, and the mechanical properties are affected by the external loading rate^1^, which induces dynamic disasters such as rock burst. Especially for coal rock with burst tendency, it is necessary to pay attention to its mechanical characteristics and failure modes. Therefore, it is of great significance for coal mine safety mining and disaster prevention to explore the influence of different loading rates on rock burst tendency.

The mechanical properties of coal rock are not only affected by the external stress state, but also related to the loading rate. This is because the change of loading rate will change the mechanical properties of the material. Therefore, the mechanical effect of rock mass under different loading rates is an important research topic in rock engineering^2^. Chong^3^ and Lajtal^4^ carried out indoor rock triaxial compression tests on rocks with strain rates of 10^–4^ s^-1^–10^–1^ s^-1^ and 3.5 × 10^–8^ s^-1^–2.5 × 10^–5^ s^-1^, respectively, indicating that the compressive strength of rocks is positively correlated with the loading rate. However, with the in-depth analysis of the mechanical properties of rock under different loading rates by many scholars, it is found that the relationship between loading rate and rock strength is not a single positive correlation, but there is a corresponding ‘critical rate’ ( If the loading rate is less than the critical rate, the strength of the rock will gradually increase with the increase of the loading rate ; if the loading rate is greater than the critical rate, the strength of the rock gradually decreases with the increase of the loading rate ). Swan et al.^5^ revealed the effect of loading rate on the triaxial compression mechanical properties of saturated shale when the strain rate was in the range of 10^–4^–240 min^-1^, and proposed the corresponding critical rate of 0.1 min^-1^. In addition, Zhang et al.^6^ also analyzed that the peak strength and elastic modulus of limestone increased sharply in the range of 5 × 10^–4^–5 × 10^–3^ mm/s and decreased in the range of 5 × 10^–3^–5 × 10^–1^ mm/s through uniaxial compression test of limestone. The damage of rock under different loading rates can be described not only by mechanical characteristics, but also by rock failure characteristics^7–10^, acoustic emission monitoring^11,12^, energy evolution characteristics^13,14^. Because the evolution law of internal energy of rock will directly reflect the damage and failure of rock under the influence of loading rate, the influence of different loading rates on rock damage and failure can be explained from the perspective of rock energy evolution.

The damage of rock is an energy-driven unstable state. Analyzing the deformation and failure mechanism of rock from the perspective of energy can better understand the nature of its failure. Many scholars have carried out experimental and theoretical studies on the energy evolution mechanism of rock deformation and failure process^15–17^, indicating that the change of loading conditions will affect the energy density inside the rock. According to the uniaxial compression test and triaxial compression test of rock, scholars have studied the rock energy characteristics at the pre-peak stage or a certain characteristic point (peak stress point, yield stress point) of rock^18–20^, and used the rock energy release rate to reflect the damage degree of rock. Based on the theory of rock energy dissipation, Xie et al.^21^ defined the concepts of releasable strain energy and unit dissipated energy, and proposed the strength criterion of rock. Aiming at the problem of dynamic failure in rock engineering, scholars have studied the energy dissipation of rock under impact and dynamic load, and analyzed that the degree of fragmentation of rock^22,23^ is positively correlated with the energy absorption per unit volume. The reason of rock unloading failure in rock triaxial unloading test is that the elastic energy accumulated by rock before unloading is released in large quantities, which leads to crack propagation^24,25^. Therefore, the failure of rock is related to the release of elastic energy. At the same time, according to the characteristics of nonlinear evolution of rock energy in the process of deformation and failure, Meng^26^ analyzed the nonlinear energy evolution mechanism of rock under lithology and loading rate, while Wang et al.^27^ analyzed the nonlinear energy evolution mechanism of limestone under uniaxial compression based on nonlinear dynamics and rock energy evolution theory, and established an energy self-promotion-inhibition model.

In summary, scholars have made a lot of achievements in the study of mechanical properties and energy evolution of rock under different loading rates. However, there are few studies on the energy evolution mechanism of coal rock under different strain rates, especially on the impact-prone coal rock. Therefore, through uniaxial compression test and triaxial compression test of impact-prone coal rock under different strain rates, the mechanical parameters and impact tendency of deformation and failure of impact-prone coal rock under different strain rates are studied, and the energy evolution mechanism and self-promotion-inhibition mechanism of deformation and failure of impact-prone coal rock under different strain rates are analyzed. The research results can provide reference for the prevention and early warning of dynamic disasters of coal and rock mass with impact tendency.

## Test scheme and mechanical characteristics analysis

### Test scheme

The test coal samples were taken from Binchang mining area, and the general/normal physical parameters are shown in Table 1. The collected coal blocks were processed into standard cylinders (diameter D = 50 mm, height H = 100 mm) by core drilling machine, cutting machine, and grinding machine to ensure that the flatness deviation of the two ends of the sample was controlled in the range of 0–0.05 mm. In order to eliminate the discreteness of the sample, the density and ultrasonic longitudinal wave velocity of the sample were screened. The impact tendency of coal rock refers to the characteristics of high accumulation of deformation energy and impact failure in coal rock mass^28^. The impact tendency of coal rock is analyzed by laboratory rock mechanics test^29^. The uniaxial compressive strength of coal rock is 13.33 MPa, the elastic energy index is 6.85, the impact energy index is 5.71, and the dynamic failure time is 649 ms. Therefore, it can be determined that the sample is a strong impact tendency coal rock.

**Table 1:** General/normal physical parameters of coal rock.

Buried depth of coal rock/m	Density /g cm^-3^	Porosity /%	Water content/%	Longitudinal wave velocity/km s^-1^
700	48.55–49.89	5.8–13.4	2.4–5.7	2.16–2.72

**Figure 1:**
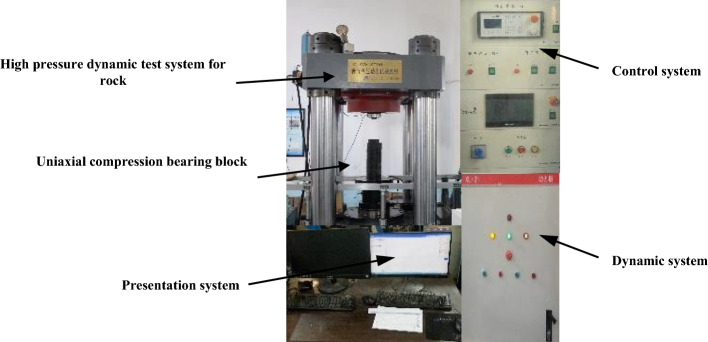
Test device diagram.

DTAW-8000 rock high pressure dynamic testing machine can be applied to rock uniaxial compression test and triaxial compression test. The whole test is controlled by computer program. The loading rate is 0.001–7 mm/s, the stress rate is 0.01–300 kN/m, the maximum loading force can reach 8000 kN, and the host stiffness is 6 GN/m. The test device is shown in Fig. 1.

In order to analyze the influence of strain rate on the mechanical characteristics and energy dissipation mechanism of coal rock, uniaxial compression test and triaxial compression test were carried out respectively. The strain rates of coal rock were 1 × 10^−5^ s^−1^, 1 × 10^−4^ s^−1^ and 1 × 10^−3^ s^−1^ respectively. Due to the more complex occurrence conditions of deep-buried coal seams, it is more authentic to study the triaxial compression coal and rock under different strain rates with similar characteristics of deep-buried engineering mining. Therefore, according to the buried depth of the coal sample, the confining pressure of the triaxial compression test is taken as 8, 12, 16 MPa, and then the influence of different strain rates on the coal and rock under the triaxial compression state is studied. In order to reduce the friction force of the contact surface between the press devices and the coal sample and reduce the end effect of the rock, a layer of vaseline with a thickness of less than 1 mm was applied to the contact surface. When the sample was installed, the sample data was measured in real time by force and displacement sensors.

### Mechanical characteristics analysis

As shown in Table 2, it can be seen that the mechanical parameters of coal rock under different strain rates under uniaxial compression and triaxial compression are different, but the mechanical characteristics of coal rock under different strain rates under triaxial compression are similar. Because the strain rate has the same influence on the mechanical characteristics of coal rock under different confining pressures, this paper will analyze the mechanical properties and energy evolution characteristics of coal rock under uniaxial and triaxial conditions (confining pressure is 12 MPa). The reduction rate in the table is the reduction ratio of rock mechanics parameters at high strain rate to the peak strength or elastic modulus of rock at low strain rate. The peak strain is the axial strain at the peak point of the rock stress–strain curve.

**Table 2:** Test results of coal rock under different strain rates.

Test	Confining pressure/MPa	Strain rate/s^-1^	Peak strength		Elastic modulus		Peak strain %
			Numerical value/MPa	Reduction rate/%	Numerical value/GPa	Reduction rate/%	
Uniaxial compression	0	1 × 10^–5^	13.33		3.29		0.45
	0	1 × 10^–4^	7.39	44.5	2.05	37.6	0.53
	0	1 × 10^–3^	4.88	33.9	1.45	29.2	0.47
Triaxial compression	8	1 × 10^–5^	22.16		3.42		0.65
	8	1 × 10^–4^	33.08	− 49.2	3.67	− 7.3	1.13
	8	1 × 10^–3^	17.84	45.9	3.16	13.8	0.57
	12	1 × 10^–5^	26.28		3.51		0.82
	12	1 × 10^–4^	40.21	− 53.1	3.99	− 13.6	1.51
	12	1 × 10^–3^	22.21	44.7	3.46	13.2	0.68
	16	1 × 10^–5^	51.45		4.98		1.32
	16	1 × 10^–4^	60.83	− 18.2	5.34	− 7.2	1.88
	16	1 × 10^–3^	40.21	33.9	4.62	13.4	0.93

**Figure 2:**
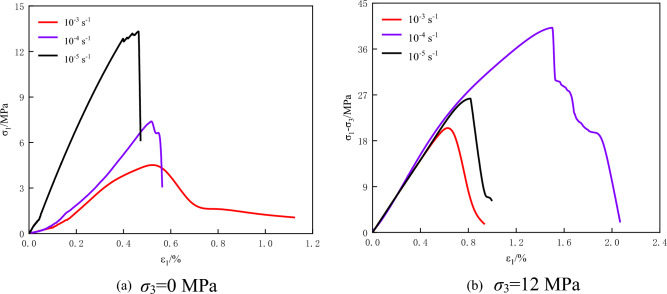
Triaxial stress–strain curves of coal rock under different strain rates.

As shown in Fig. 2, the strain rate will affect the strength and deformation characteristics of the sample, which is mainly reflected in the peak strength, elastic modulus, and peak strain of the sample. Under uniaxial compression, with the increase of strain rate, the peak strength and elastic modulus of coal rock decrease, but the reduction rate gradually slows down, and the peak strain increases first and then decreases. When the strain rate is 1 × 10^–5^ s^-1^, the post-peak stress–strain curve of coal and rock produces a ‘sudden decreasing’ stress drop, which has a small residual stress. When the strain rate is 1 × 10^–4^ s^-1^ and 1 × 10^–3^ s^-1^, the initial cracks close rapidly, and the whole coal rock is unstable and destroyed, which appears obvious brittle characteristics. Under triaxial compression, the peak strength, elastic modulus, and peak strain of coal rock increase first and then decrease with the increase of the loading rates. Among them, the compaction stage of coal rock gradually decreases, the elastic and plastic stages are prolonged, and the final failure has certain ductility characteristics, indicating that the confining pressure weakens the initial fracture and other structures on the strength of coal rock.

### Impact tendency analysis of coal rock under different strain rates

The stress environment in the mining area was the external condition that induced rock burst disasters, and the impact tendency of coal rock mass was the essential factor. The change of advance speed of mining would affect the loading mode and stress path of coal rock, and further impact its failure mode. In order to investigate the effect of strain rate on the impact tendency of coal rock, this paper uses the brittleness index modification index (BIM) to analyse the impact tendency of coal rock under different strain rates.

BIM index is to simplify the unloading curve to a straight line passing through the peak point with E50 as the slope, which is expressed as the ratio of the total energy E stored in the coal rock to the elastic energy Ee. When the elastic energy stored in the coal rock approached the total energy, the value of BIM was close to 1. At this time, the energy input from the outside was stored as elastic energy, and the coal rock energy was released instantly after the peak, and rock burst occurred. Therefore, the larger the BIM value, the lower the impact tendency of coal rock. M.Aubertin^30^ divided the impact tendency according to the BIM value, as shown in Table 3.

**Table 3:** Impact tendency evaluation based on BIM.

BIM	Burst tendency
1.00 ≤ BIM ≤ 1.20	High
1.20 < BIM ≤ 1.50	Medium
BIM > 1.50	Low

Table 4 lists the BIM values of coal rock at different strain rates. With the increase of strain rate, the BIM value decreases first and then increases. When the strain rate is 1 × 10^–5^ s^-1^, the impact tendency evaluation of coal rock is medium. When the strain rate increases to 1 × 10^–4^ s^-1^, the impact tendency evaluation of coal rock is high. When the strain rate is 1 × 10^–3^ s^-1^, the impact tendency evaluation of coal rock is low. Therefore, the impact tendency is affected by the strain rate, and increases first and then decreases. With the increase of strain rate, the accumulation ability of elastic energy of coal rock increases first and then decreases. It shows that the impact tendency of coal rock will increase first and then decrease with the increase of strain rate, and then change the elastic energy accumulation ability of coal rock.

**Table 4:** BIM index of coal and rock at different strain rates.

Strain rate /s^-1^	A_1_/kJ m^-3^	A_2_/kJ m^-3^	BIM
1 × 10^–5^	25.72	34.87	1.35
1 × 10^–4^	15.87	16.89	1.06
1 × 10^–3^	7.61	13.86	1.82

## Energy evolution law of coal rock with burst tendency

### Energy dissipation theory of rock

The deformation and failure of coal rock are accompanied by four energy conversion forms: energy input, energy accumulation, energy dissipation and energy release. The total energy input from the outside is mainly converted into elastic energy and dissipative energy^31,32^. Elastic strain energy is the energy stored in the process of reversible deformation of coal rock mass, which eventually leads to the deformation and failure of coal rock by accumulating energy. The dissipated energy is the energy lost by coal and rock during irreversible deformation (plastic deformation, damage, friction, and thermal radiation).

**Figure 3:**
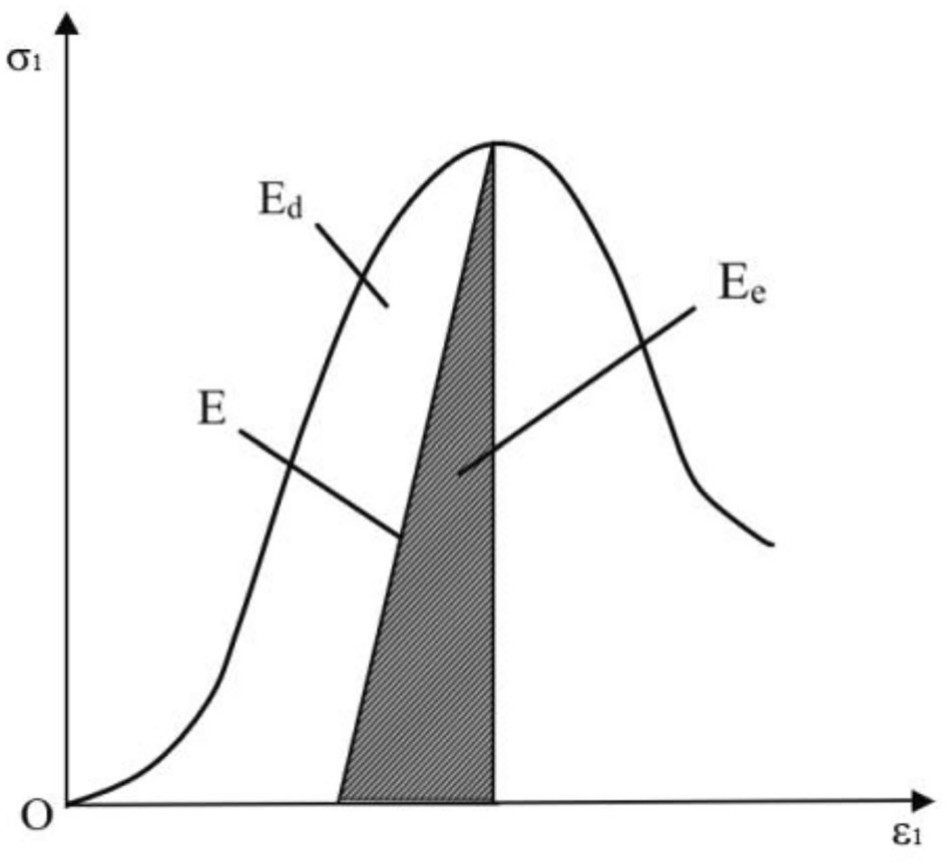
Relationship between dissipated energy and elastic strain energy in coal rock.

The process of energy dissipation is a dynamic process of continuous development, extension, derivation, weakening and disappearance of the internal structure of coal rock^20,33^. According to the first law of thermodynamics.

1 $$ E = E_{e} + E_{d} $$

where E is the total strain energy input from the outside, kJ/m^3^; Ee is the elastic strain energy during coal rock deformation, kJ/m^3^; Ed is the dissipated energy during coal rock deformation, kJ/m^3^. Figure 3 shows the relationship between dissipated energy and elastic strain energy in coal rock.

According to the energy analysis of the coal rock, the energy in each part of the coal rock is:

2 $$ E = \mathop \smallint \limits_{0}^{{\varepsilon_{1} }} \sigma_{1} d\varepsilon_{1} + \mathop \smallint \limits_{0}^{{\varepsilon_{2} }} \sigma_{2} d\varepsilon_{2} + \mathop \smallint \limits_{0}^{{\varepsilon_{3} }} \sigma_{3} d\varepsilon_{3} $$

3 $$ E_{e} = \frac{1}{2}\sigma_{1} \varepsilon_{1}^{e} + \frac{1}{2}\sigma_{1} \varepsilon_{2}^{e} + \frac{1}{2}\sigma_{3} \varepsilon_{3}^{e} $$

where σ_1_, σ_2_ and σ_3_ are the principal stresses of coal rock; ε_1_^e^, ε_2_^e^ and ε_3_^e^ are the elastic strains along the principal stress direction.

Under the condition of uniaxial compression, σ_2_ and σ_3_ are both 0, so the total strain energy and elastic strain energy of coal rock are simplified as:

4 $$ E = \smallint \sigma_{1} d\varepsilon_{1} = \mathop \sum \limits_{i = 1}^{n} \frac{1}{2}\left( {\sigma_{1i + 1} + \sigma_{1i} } \right)\left( {\varepsilon_{1i + 1} - \varepsilon_{1i} } \right) $$

5 $$ E_{e} = \frac{1}{2}\sigma_{1} \varepsilon_{1}^{e} = \frac{{\sigma_{1}^{2} }}{{2E_{u} }} $$

where σ_1i_ and ε_1i_ are the stress and strain values on the principal stress–strain curve. In the calculation of elastic strain energy per unit of coal rock, the elastic mod-ulus E_0_ can approximately replace E_u_^34,35^. The error caused by the replacement is generally within 5%^36^. Thus, the elastic strain energy E_e_ can be approximately given by

6 $$ E_{e} \approx \frac{{\sigma_{1}^{2} }}{{2E_{0} }} $$

Under the conventional triaxial condition, it is assumed that the coal rock mass is isotropic, and the circumferential elastic strain energy is relatively small and negligible compared with the axial elastic strain energy^37^. Therefore, the elastic strain energy in coal rock can be calculated according to Formula (6). The total strain energy E in coal is:

7 $$ E = \sum\limits_{i = 1}^{n} \frac{1}{2} \left( {\sigma_{1i} + \sigma_{1i - 1} } \right)\left( {\varepsilon_{1i} - \varepsilon_{1i - 1} } \right) - 2\sum\limits_{i = 1}^{n} {\sigma_{3} \left( {\varepsilon_{3i} - \varepsilon_{3i - 1} } \right)} . $$

### Energy evolution law

Based on the theory of rock energy dissipation, the experimental data are substituted into Formulae (1) (4) (6) (7) to calculate the total energy E, elastic energy E_e_, dissipated energy E_d_, elastic energy ratio and dissipated energy ratio in the deformation and failure of coal rock under uniaxial compression and triaxial compression. The energy evolution curves of coal rock with impact tendency under different strain rates are shown in Fig. 5.

**Figure 4:**
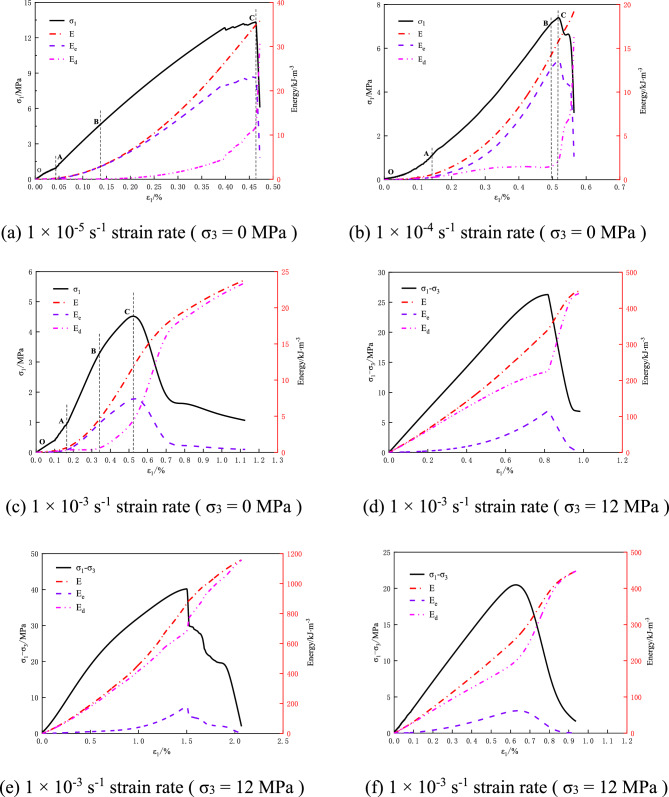
Energy evolution curves of coal rock under different strain rates.

It can be seen from Fig. 4a–c that the total energy, elastic energy, and dissipation energy show different evolution characteristics during the deformation and failure process of impact-prone coal rock under uniaxial compression. The elastic energy increases nonlinearly before the peak value. When the energy storage limit is reached, the elastic energy stored in the coal rock is suddenly released due to the failure of coal rock. The dissipation energy increases slowly in the process of initial fracture compaction. The dissipation energy in the elastic stage is in a stable state, and the dissipation energy in the plastic stage will continue to increase until the dissipation energy of coal rock failure increases suddenly. Due to the large strain rate at high strain rate of 1 × 10^–3^ s^−1^, the internal structure of coal rock is not completely destroyed, and it still has a certain bearing capacity after the peak. With the further development of deformation and failure, the internal structure of coal rock is damaged, and coal rock produces obvious plastic deformation.

As shown in Fig. 4d–f, compared with the energy evolution of coal rock under uniaxial compression, the change trend of total energy, elastic energy, and dissipation energy of coal rock under triaxial compression is similar. However, due to the action of confining pressure, the initial cracks in coal rock are quickly compacted and the new cracks expand rapidly, so the duration of coal rock compaction stage and elastic stage is very short. After entering the plastic stage, with the increase of load, the dissipated energy in coal rock is much higher than the elastic energy. Therefore, the energy dissipation curves of coal and rock with different strain rates under uniaxial compression are only divided into stages in this paper.

**Figure 5:**
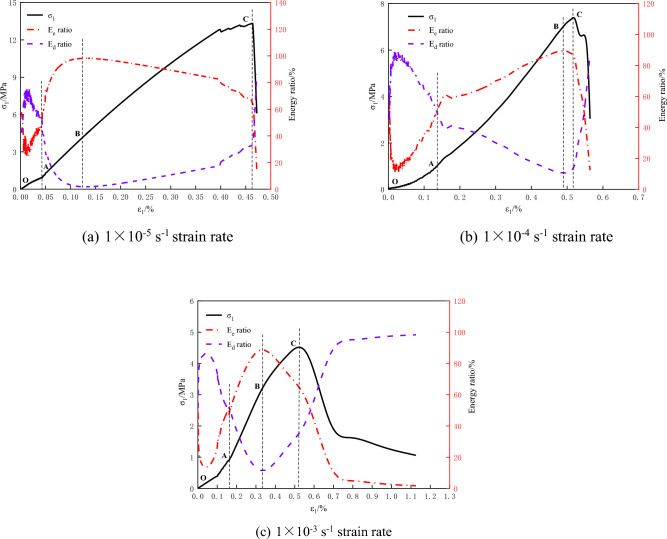
Internal energy ratio curve of uniaxial compression coal rock under different strain rates.

The proportion of elastic energy and dissipated energy in the deformation and failure process of coal rock with impact tendency reflects the internal energy structure of coal rock. As shown in Fig. 5, the elastic energy ratio curve of coal rock with different strain rates under uniaxial compression is ‘S’ type. Therefore, according to the energy ratio of coal rock with different strain rates under uniaxial compression, the energy evolution of coal rock with impact tendency can be divided into four stages: initial energy damage stage, energy hardening stage, energy softening stage and failure stage.

#### Initial energy damage stage (from O to A)

The dissipation energy increases nonlinearly with the increase of deformation, and the proportion of dissipation energy Ed in coal rock is greater than that of elastic energy Ee, which is mainly caused by the closure and friction of microcracks in coal rock. The total energy and elastic energy also increase with the increase of deformation, and with the closure of micro cracks, the growth rate of elastic energy increases gradually, and finally the coal rock enters the next stage (energy hardening stage) after the dissipation energy is equal to the elastic energy.

#### Energy hardening stage (from A to B)

After the micro-fractures in coal rock are completely closed, the total energy and elastic energy increase with the same trend with the increase of coal rock deformation, and the growth rate is greater than the dissipation energy growth curve. The dissipation energy curve is approximately horizontal, and the elastic performance is greater than the dissipation energy in the whole process (the proportion of elastic energy Ee is greater than the proportion of dissipation energy Ed). The total energy input at this stage is basically stored in the coal rock, and the elastic properties of the coal rock are continuously accumulated, and the energy dissipation is less.

#### Energy softening stage (from B to C)

As the proportion of elastic energy in coal rock reaches the peak, the total energy and elastic energy in coal rock increase with the increase of deformation, but the growth rate of elastic energy decreases gradually. At this stage, part of the total energy input continues to be stored in the form of elastic energy, and part of it is rapidly released by friction, expansion, and new cracks. Therefore, the growth rate of dissipated energy is gradually increasing (the proportion of dissipated energy increases). The deformation ability of coal rock is obviously enhanced, and new microcracks are generated and extended inside the coal rock. The generation of these cracks is bound to cause more energy dissipation.

#### Failure stage (after point C)

When the peak strength is reached, the rock enters the failure stage. At this stage, the dissipation energy evolution curve increases sharply, while the elastic energy evolution curve decreases sharply. This is due to the rapid expansion and penetration of internal cracks in coal rock, resulting in the loss of bearing capacity of coal rock, and the elastic energy accumulated in coal rock is quickly released in the form of dissipated energy, resulting in a sharp increase in the dissipation energy curve.

Under uniaxial compression, when the strain rate is 1 × 10^–4^ s^−1^ (Fig. 5), the dissipated energy in the energy hardening stage (AB stage) increases slowly first and then gradually stabilizes. The possible reason was that there were new cracks in the coal rock at the early stage of energy hardening, causing an increase in the dissipated energy, and the dissipated energy became stable after the cracks were closed. As a result, the proportion of dissipated energy in energy hardening stage (A to B) in Fig. 5 B rose dramatically. In Reference^38^, the energy evolution of rock is divided into three stages ac-cording to the energy ratio characteristics. In this paper, by contrast, the energy evolution was divided into four stages: initial energy damage stage, energy hardening stage, energy softening stage, and failure stage. This helps to better reflect the internal energy mechanism of burst-prone coal rock during deformation and failure, so as to investigate the energy evolution law of burst-prone coal rock under different strain rates.

### Analysis of peak point energy index under different strain rates

The strain rate will affect the energy index of the peak point of the impact tendency coal rock. As shown in Table 5, with the increase of strain rate, the total energy and elastic energy of coal rock at peak point under uniaxial compression continue to decrease. This is because there are many cracks in the impact-prone coal rock itself. With the increase of strain rate, the stress concentration at the crack is more obvious, which leads to the expansion and penetration of more cracks. The energy required for specimen failure is decreasing, and the elastic energy stored in coal rock will also decrease. The proportion of elastic energy at the peak point of coal rock increases first and then decreases with the increase of strain rate, indicating that the elastic energy accumulation ability of impact coal rock increases first and then decreases with the increase of strain rate.

**Table 5:** Energy index of coal rock peak point at different strain rates.

Confining pressure/MPa	Strain rate/s^−1^	Total energy /kJ m^−3^	Elastic energy/kJ m^−3^	Proportion of elastic energy/%	Dissipated energy/kJ m^−3^	Proportion of dissipated energy/%
0	1 × 10^–5^	34.87	23.42	67.16	11.45	32.84
	1 × 10^–4^	16.89	14.12	83.59	2.77	16.40
	1 × 10^–3^	13.86	8.52	61.47	5.33	38.43
12	1 × 10^–5^	341.8	117.1	34.25	224.8	65.75
	1 × 10^–4^	874.3	188.4	21.55	685.8	78.45
	1 × 10^–3^	287.6	71.26	24.78	216.3	75.22

Compared with uniaxial compression, the energy index of coal peak point varies with different strain rates under triaxial compression. With the increase of strain rate, the total energy, elastic energy, and dissipated energy of at the peak point of coal increase first and then decrease. It shows that when the strain rate reaches 1 × 10^–4^ s^−1^, the coal rock burst risk is the highest in the coal rock mass with high buried depth.

## Energy self-promotion-inhibition mechanism of burst-prone coal rock

The external input energy is transformed into elastic energy accumulated inside the rock mass. This effect is known as strain hardening effect. Accordingly, what suppresses this effect is the strain softening effect. There are two mechanisms (energy accumulation mechanism and energy dissipation mechanism) in the pre-peak energy conversion process of burst-prone coal rocks, which are driven by the pre-peak strain hardening deformation mechanism of coal rock^39^. According to the analysis of microscopic deformation mechanism of rock^40^, the total energy of the rock at time t is U, at which there are m types of strain hardening effects and n types of strain softening effects The energy transferred by each effect at time t is U_i_ and U_k_, respectively, the total mechanical energy input from the outside is U_c_, and the energy released from the rock is U_t_.

8 $$ U = \mathop \sum \limits_{i = 1}^{m} U_{i} + \mathop \sum \limits_{k = 1}^{n} U_{k} $$

Assuming that there was only one strain hardening effect and strain softening effect in the microscopic deformation process, then they two effects can be expressed as

9 $$ \frac{{dU_{i} }}{{d_{t} }} = \left[ {{\text{a}}_{i0} \left( {U_{c} - U_{i0} } \right) - {\text{a}}_{i} U_{i} + {\text{a}}_{ik} U_{k} } \right]U_{i} $$

10 $$ \frac{{dU_{k} }}{{d_{t} }} = \left[ {{\text{b}}_{k0} \left( {U_{i} - U_{k0} } \right) - \left( {b_{k1} - b_{k2} } \right)U_{k} - {\text{b}}_{k3} U_{t} } \right]U_{k} $$

where U_i0_ is the lowest activation energy of the i-th hardening mechanism; U_k0_ is the lowest activation energy of the k-th softening mechanism; a_i0_ is the promotion coefficient of strain hardening; ai is the inhibition coefficient of the i-th strain hardening mechanism; a_ik_ is the coefficient of converting U_i_ into heat energy and internal energy; b_k1_ is the self-competition coefficient of the k-th strain softening mechanism; b_k2_ is the self-promotion coefficient of the k-th strain softening mechanism; b_k3_ is the energy diffusion coefficient.

The strain hardening mechanism converted the external input energy into elastic energy, but the accumulation of a large amount of elastic energy was also affected by the self-inhibition effect. Since the temperature of the indoor test changed slowly, it is assumed that the thermal energy loss in the coal specimen was not considered. So Eq. (9) can be rewritten as:

11 $$ \frac{{dU_{e} }}{{d_{t} }} = \left[ {{\text{a}}_{e0} \left( {U_{ec} - U_{i0} } \right) - {\text{a}}_{e} U_{e} } \right]U_{e} $$

where U_e_ is the elastic energy, U_i0_ is the activation value of elastic energy, U_ec_ is the energy input from the outside to promote the accumulation of elastic energy, a_e0_ is the promotion activation coefficient, and a_e_ is the inhibition coefficient. Therefore, the elastic energy of coal rock mass can be expressed as

12 $$ U_{e} = \frac{b}{{{\text{a}}_{e} + e^{{{\text{a}}_{e} k - bt}} }} $$

where b = a_e0_(U_ec_ − U_i0_), and k is a constant.

**Figure 6:**
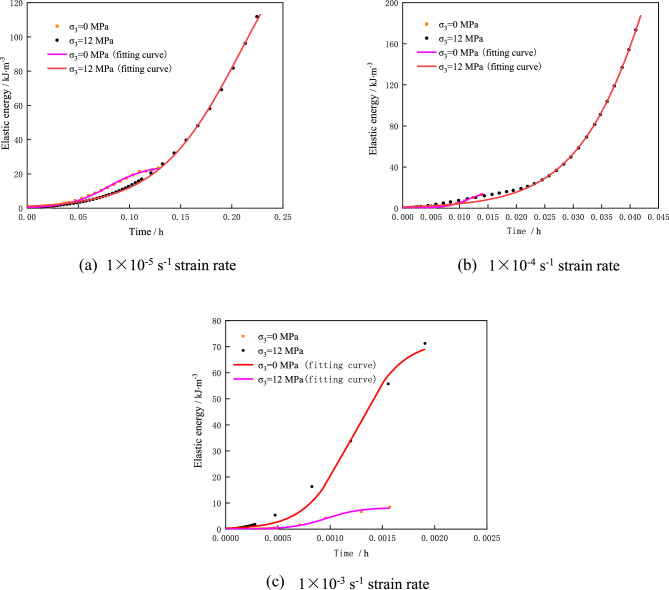
Fitting curves of elastic energy of coal rock under different strain rates.

Equation 12 is the self-promotion-inhibition evolution model of pre-peak energy of burst-prone coal rock. By fitting the pre-peak elastic energy of the burst-prone coal rock under different strain rates, the elastic energy inhibition coefficient ae and the promotion coefficient k can be obtained, so as to analyze the elastic energy variation characteristics of the burst-prone coal rock under different strain rates. The uniaxial and triaxial compression test data of coal rock under different strain rates (1 × 10^-5^ s^-1^, 1 × 10^-4^ s^-1^ and 1 × 10^-3^ s^-1^) are fitted according to the model in Eq. 12. Thus, the nonlinear fitting curve of pre-peak elastic energy is obtained, as showed in Fig. 6. The corresponding elastic energy fitting parameters ae and b are shown in Table 6.

**Table 6:** Coal rock fitting parameters under different strain rates.

Confining pressure/MPa	Strain rate/s^-1^	Inhibition coefficient (*a*_*e*_)		Promotion coefficient (*b*)		*R*^2^
		Numerical value	Amplification	Numerical value	Amplification	
0	1 × 10^–5^	2.04		51.25		0.997
	1 × 10^–4^	27.86	12.65	495.5	8.66	0.997
	1 × 10^–3^	741.78	25.62	6124.2	11.35	0.992
12	1 × 10^–5^	0.13		24.3		0.998
	1 × 10^–4^	0.86	5.61	128.2	4.27	0.998
	1 × 10^–3^	61.52	303.6	4446.2	33.68	0.992

According to the fitting parameter R^2^, the self-promotion-inhibition evolution model of pre-peak energy of coal rock fits well with the experimental results, which can truly reflect the self-promotion-inhibition evolution process of pre-peak energy of coal rock. Herein, a_e_ is the inhibition coefficient of elastic energy, and b is the promotion coefficient of elastic energy.

It can be seen from Fig. 6 that the promotion effect of elastic properties in coal rock is greater than the inhibition effect of elastic energy. Therefore, the elastic energy accumulates rapidly in coal rock, which further leads to the failure of coal rock. When the strain rate is 1 × 10^–5^ s^-1^, the internal elastic strain energy of coal rock accumulates slowly in the rock sample, so the inhibition effect and promotion effect of elastic energy of coal rock are low at low strain rate of 1 × 10^–5^ s^-1^. With the increase of strain rate, the rate of elastic energy generation in coal rock will gradually increase, and the faster elastic energy accumulation will lead to the enhancement of elastic energy inhibition effect. Therefore, the elastic energy promotion coefficient and inhibition coefficient increase with the increase of strain rate, and the increase of elastic energy inhibition coefficient is greater than that of promotion coefficient.

In summary, the pre-peak elastic energy promotion coefficient of impact prone coal rock is greater than the inhibition coefficient. With the increase of strain rate, the elastic energy promotion coefficient and inhibition coefficient of coal rock mass also increase, and the promotion coefficient increases greatly, indicating that the increase of strain rate will promote the generation of elastic energy inside coal rock.

## Conclusions

In this paper, the uniaxial compression test and triaxial compression test of coal rock with burst tendency are used to study the mechanical properties of coal rock with burst tendency under different strain rates, the change law of burst tendency, the law of energy evolution and the self-promotion-inhibition mechanism of pre-peak energy. The main conclusions are as follows:

1. The peak strength and elastic modulus of coal rock with impact tendency in uniaxial compression test decrease with the increase of strain rate, while the peak strength and elastic modulus of coal rock in triaxial compression test increase first and then decrease with the increase of strain rate. According to the impact tendency energy index BIM, it is determined that the impact tendency of coal rock under uniaxial compression will increase first and then decrease with the increase of strain rate.
2. In the uniaxial compression test, according to the change law of the energy proportion of the coal rock with impact tendency, the evolution of the load energy of the coal rock with impact tendency is divided into four stages: initial energy damage stage, energy hardening stage, energy softening stage and failure stage.
3. The conversion rate of dissipated energy in coal rock with impact tendency at peak point is positively correlated with strain rate. Under uniaxial compression, the total energy and elastic energy of coal rock with peak impact tendency decrease with the increase of strain rate, and the change of strain rate will affect the elastic energy accumulation force of coal rock. In addition, with the increase of strain rate under triaxial compression, the total energy, elastic energy, and dissipated energy of coal rock at the peak point increase first and then decrease, which shows that the impact tendency of coal rock has the highest impact risk when the strain rate is 1 × 10^–4^ s^-1^ under confining pressure. the action of confining pressure.
4. A pre-peak energy self-promotion-inhibition evolution model of impact prone coal rock is established. The elastic energy promotion coefficient of impact prone coal rock is much larger than the inhibition coefficient. With the increase of strain rate, the promotion coefficient and inhibition coefficient of coal rock elastic energy will gradually increase, and the promotion coefficient will increase greatly. Therefore, the increase of strain rate will promote the generation of elastic energy inside coal rock.

## Data Availability

The data used to support the findings of this study are available from the corresponding author upon request.
